# Pheochromocytoma and primary hyperparathyroidism: a very rare association in a neurofibromatosis type 1 patient unmasked by Takotsubo syndrome

**DOI:** 10.1530/EDM-25-0109

**Published:** 2026-04-29

**Authors:** Nuno Faria, Valentim Lopes, Tamara Pereira, Olga Azevedo, António Lourenço, Adriana De Sousa Lages, Catarina Martins Machado

**Affiliations:** ^1^ Endocrinology Department, Hospital de Braga, Braga, Portugal; ^2^ Cardiology Department, Hospital Senhora da Oliveira, Guimarães, Portugal; ^3^ Faculty of Medicine, Universidade de Coimbra, Coimbra, Portugal

**Keywords:** neurofibromatosis type 1, pheochromocytoma, primary hyperparathyroidism, Takotsubo syndrome, breast carcinoma

## Abstract

**Summary:**

Neurofibromatosis type 1 is an autosomal dominant disease characterized by cutaneous, bone, and neurocognitive manifestations and an increased risk of neoplasms – more frequently cutaneous neurofibromas and optic gliomas and more rarely pheochromocytomas/paragangliomas, solitary parathyroid adenomas with primary hyperparathyroidism, and breast carcinomas. We present a case of a 48-year-old woman with an unexpected presentation of Takotsubo syndrome that culminated in a synchronous diagnosis of pheochromocytoma, primary hyperparathyroidism, and invasive breast carcinoma (pN2(R0)N1) in the context of previously unknown neurofibromatosis type 1. The patient underwent simultaneous right adrenalectomy and lower parathyroidectomy nearly one month after left lumpectomy with axillary ganglion dissection. No complications were reported after adequate alpha- and beta-adrenergic blockade before or during both surgical interventions. Furthermore, after definitive treatment of catecholamine hypersecretion, postoperative chemoradiotherapy was required. Our case highlights the importance of differential diagnosis with multiple endocrine neoplasia type 2 and the relevance of multidisciplinary teams in the management of complications associated with neurofibromatosis type 1, namely those related to neoplasms.

**Learning points:**

- Neurofibromatosis type 1 (NF1) is an autosomal dominant disease characterized by cutaneous, bone, and neurocognitive manifestations and an increased risk of neoplasms, namely pheochromocytomas/paragangliomas, solitary parathyroid adenomas, and breast carcinomas.
- Pheochromocytomas/paragangliomas (PPGLs) occur in up to 15% of patients. Pheochromocytomas can rarely present as Takotsubo syndrome (TS), and the occurrence of TS in NF1 patients is even rarer.
- In addition, the co-occurrence of PPGLs and primary hyperparathyroidism (PHPT) in NF1 is very infrequent, mimicking MEN 2A syndrome.
- This is the first case described in the literature of a patient with NF1 and a synchronous diagnosis of pheochromocytoma, PHPT, and breast carcinoma, highlighting the relevance of a high level of suspicion of all the nosological entities that can be associated with NF1 in order to allow an early diagnosis.

## Background

Neurofibromatosis type 1 (NF1) is an autosomal dominant disease resulting from a mutation in the tumor suppressor gene NF1, which encodes the protein neurofibromin. The disease is characterized by the presence of cutaneous (café-au-lait spots and axillary/inguinal freckling), osseous (sphenoid dysplasia and scoliosis), neoplastic (optic and cerebral tract gliomas, cutaneous and plexiform neurofibromas, malignant peripheral nerve sheath tumors, and pheochromocytomas/paragangliomas), and neurocognitive (learning difficulties and epilepsy) manifestations, some of which are progressive and lead to significant morbidity or mortality (1).

Despite being an autosomal dominant hereditary disease, in up to 50% of cases, it arises from a *de novo* mutation in individuals with no family history of NF1 (2).

Pheochromocytomas/paragangliomas (PPGLs) occur in up to 15% of patients, being one of the most common endocrine neoplasms associated with NF1 (3). Pheochromocytomas can rarely present as Takotsubo syndrome (TS), and the occurrence of TS in NF1 patients is even rarer (4, 5). The risk of breast cancer in NF1 is also increased, with the risk estimated to be more than 10 times higher than in the general population (6).

However, the occurrence of primary hyperparathyroidism (PHPT) in patients with NF1 is uncommon, and the co-existence of PPGL and PHPT is even less commonly reported, mimicking the classical multiple endocrine neoplasia type 2 (MEN2) (7).

## Case presentation

A 48-year-old woman with a history of uncontrolled hypertension, active smoking, dyslipidemia, newly diagnosed type 2 diabetes mellitus, and nephrolithiasis had been suffering from multiple episodes of headache, diaphoresis, and palpitations, labeled as panic attacks, for the past 10 years. She had an unremarkable family history.

**Figure 1:**
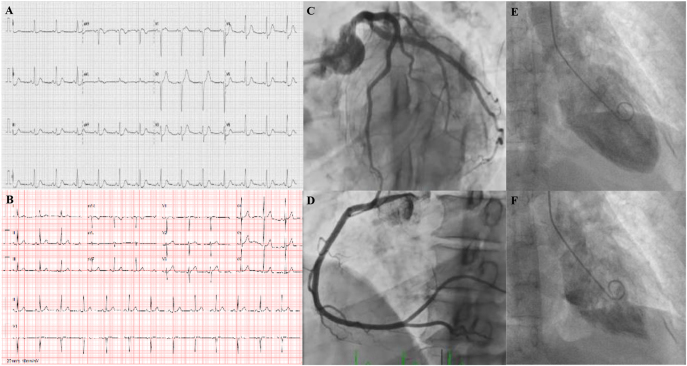
(A) Admission ECG showing sinus rhythm with HR of 81 bpm, ST-segment elevation in aVR and V1 and diffuse depression of ST-segment in inferolateral wall, with a maximum of 2 mm. (B) Pre-discharge ECG exhibiting the absence of repolarization changes. (C and D) Admission coronary angiography showing the absence of epicardial coronary disease. (E and F) Ventriculography showing akinesia of the middle and basal segments of all ventricular walls and apex hypercontractility in diastole (E) and systole (F).

One morning, she was admitted to the emergency department due to persistent oppressive chest pain, which woke her up suddenly at 07:00 h, with irradiation to the left upper limb, associated with an episode of vomiting. On admission, she was hypertensive (165/87 mmHg), and an electrocardiogram (ECG) showed ST-segment elevation in aVR and V1 and diffuse depression of ST-segment, with a maximum of 2 mm in the inferolateral wall (Fig. 1A). Emergency transthoracic echocardiography revealed a left ventricular systolic function in the lower limit of normality due to basal and mid-segment akinesia of anterior, anterolateral, and inferolateral walls and apex hypercontractility. Administration of intravenous isosorbide dinitrate normalized blood pressure but had no effect on chest pain. A loading dose of aspirin combined with ticagrelor, unfractionated heparin, and rosuvastatin was also administered, and emergent coronary angiography excluded epicardial coronary disease (Fig. 1C and D). Ventriculography confirmed the akinesia of the basal and mid segments of all ventricular walls and apex hypercontractility, suggesting a reverse TS (Fig. 1E and F).

## Investigation

**Figure 2:**
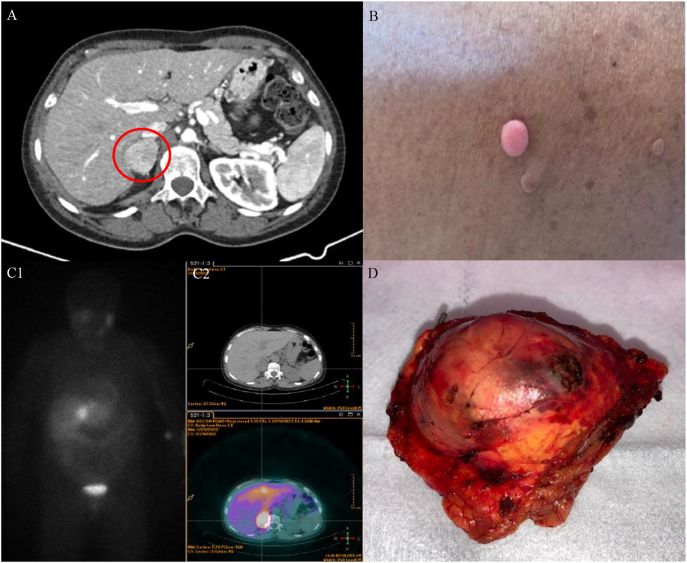
(A) Thoracoabdominopelvic computed tomography showing a contrast-enhanced heterogeneous nodule in the right adrenal gland, with dimensions of 38 × 36 mm, suggestive of pheochromocytoma. (B) Café-au-lait spots and neurofibromas scattered over the abdomen. (C1 and C2) Scintigraphy with I-123 MIBG (C1) and PET-CT with F-DOPA (C2) revealing uptake of the radiopharmaceutical in the right adrenal gland, without lesions in other locations. (D) Surgical specimen of the right adrenalectomy with evidence of a nodule of 38 mm in diameter corresponding to the pheochromocytoma.

Admission laboratory investigation showed leukocytosis and evidence of myocardial injury (troponin I of 774 ng/mL, normal <0.045 ng/mL). Few hours after admission to the cardiac intensive care unit, she became diaphoretic, with intermittent headaches, associated with paroxysmal hypertension and sinus tachycardia. Due to suspicion of an autonomous production of catecholamines as a possible etiology for this clinical scenario, plasma and urinary epinephrine, norepinephrine, and metanephrines were collected and the beta-blocker was stopped. Plasmatic epinephrine, metanephrine, and normetanephrine were significantly elevated (Table 1). A thoracoabdominopelvic computed tomography (TAP-CT) showed a contrast-enhanced heterogeneous nodule in the right adrenal gland, with dimensions of 38 × 36 mm, suggestive of pheochromocytoma (Fig. 2A).

**Table 1:** Value of plasmatic and urinary catecholamines and metanephrines after the diagnosis of Takotsubo syndrome.

Parameter	Result	Reference value
Plasma		
Epinephrine, pg/mL	322 (5 × ULN)	<60
Norepinephrine, pg/mL	635	<650
Dopamine, pg/mL	10	<30
Metanephrine, pg/mL	842 (13 × ULN)	<65
Normetanephrine, pg/mL	1,517 (8 × ULN)	<196
Urine (volume = 1,950 mL/24 h)		
Epinephrine, pg/mL	502 (28 × ULN)	<18
Norepinephrine, pg/mL	611 (8 × ULN)	<76
Dopamine, pg/mL	341	<390
Total metanephrine, pg/mL	15,318 (20 × ULN)	<785
3-Methoxytyramine, μg/mL	549 (1.3 × ULN)	<434

ULN, upper limit of normality.

Simultaneously, on the physical examination, the patient presented a tanned skin with several café-au-lait spots and neurofibromas scattered over the abdomen and back, highly suggestive of NF1, later confirmed by genetic testing that detected a pathogenic variant in heterozygosity in the *NF1* gene – c.2041C>T (p.Arg681*) – and excluded mutations in the *RET* gene (Fig. 2B).

**Figure 3:**
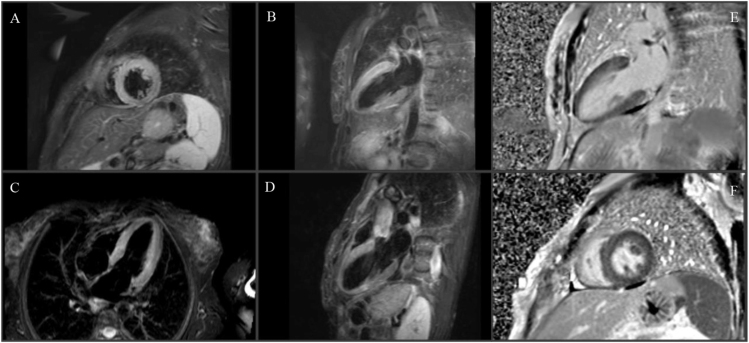
(A, B, C, D) T2-weighted images (STIR) showing hypersignal more evident in the basal and middle segments of the left ventricular anterior and anterolateral walls. (E and F) High-resolution late gadolinium enhancement (LGE) images showing no areas suggestive of the presence of myocardial necrosis or fibrosis.

The case was discussed with the endocrinology department of a tertiary center, and due to the persistence of paroxysms, the patient started alpha-adrenergic blockade with phenoxybenzamine, with clinical improvement. There were no significant changes on the ECG on the following days, with resolution of the repolarization changes (Fig. 1B). The peak troponin I level was 6,755 ng/mL on the first day of hospitalization, with a progressive fall and normalization. Five days after admission, cardiac magnetic resonance imaging was performed, revealing diffuse myocardial hypersignal on T2-weighted (STIR) images, predominantly in the basal and middle segments of the left ventricle, more pronounced in the anterior and anterolateral walls (Fig. 3A, B, C, D), with no late gadolinium enhancement (Fig. 3E and F). These findings are consistent with myocardial edema, supporting an acute reversible injury pattern typical of TS.

The patient was discharged under phenoxybenzamine 10 mg twice a day and was observed in the endocrinology consultation two weeks later. In the first consultation, she reported improvement in the intensity and frequency of paroxysms but mentioned episodes of dizziness while standing. On physical examination, orthostatic hypotension was excluded, so the phenoxybenzamine dose was maintained and beta-adrenergic blockade with propranolol 10 mg once a day was initiated. She underwent a body scintigraphy with I-123 MIBG and 18F-DOPA-PET/CT that confirmed hyperuptake in the right adrenal gland and excluded metastases or synchronous tumors (Fig. 2C).

Given the previous history of nephrolithiasis and the subjective notion of polyuria and polydipsia, calcium and phosphate metabolism analytical assessment was collected, which revealed a hypercalcemic state secondary to PHPT (Table 2).

**Table 2:** Calcium and phosphate metabolism revealing hypercalcemic primary hyperparathyroidism.

Calcium and phosphate metabolism	Result	Reference value
PTH, pg/mL	200.85	18.5–88
Total calcium, mg/dL	12.2	8.3–10.6
Corrected calcium, mg/dL	12.1	8.3–10.6
Phosphate, mg/dL	2.3	2.5–5.1
25-Hydroxyvitamin D, ng/mL	31	>30
Albumin, g/dL	4.1	3.4–5.4
Alkaline phosphatase, IU/L	95	44–147
Calcitonin, pg/mL	2	<10

PTH, parathyroid hormone.

Taking into account the clinical and analytical findings compatible with the diagnosis of hypercalcemia secondary to PHPT, a cervical ultrasound was performed. In addition to a multinodular goiter, it showed a hypoechogenic nodule, with intranodular blood flow, measuring 16 × 13 × 25 mm, located posteriorly to the lower third of the right thyroid lobe, suggestive of an adenoma of the right inferior parathyroid gland. The sestamibi scintigraphy exhibited an increased radiopharmaceutical uptake in the topography described. The patient also underwent a dual-energy X-ray absorptiometry that revealed severe osteoporosis at the lumbar spine (T-score of −4.3), femoral neck (T-score of −2.5), and radius (T-score of −3.5), for which treatment with alendronate/vitamin D 70 mg/2,800 IU once a week was initiated.

However, two months after admission to the emergency department, the patient noticed a nodule in the left breast during self-palpation and ultrasound evaluation revealed a heterogeneous nodular formation with irregular contours corresponding to a cluster of microcalcifications, classified as BI-RADS 4 on the mammogram later performed. The lesion’s biopsy revealed an invasive carcinoma without a specific type, with triple positivity for estrogen and progesterone receptors and HER2. At this moment, we were facing an invasive breast carcinoma, clinically cT2N0M0, requiring to be promptly treated prior to the definitive treatment of the pheochromocytoma and PHPT, with clear perioperative and anesthetic implications. The patient underwent, under alpha- and beta-adrenergic blockade, a left lumpectomy and axillary ganglion dissection due to the evidence, in a sentinel lymph node biopsy, of metastasis in 3 axillary ganglia. Then, the tumor was reclassified as pN2(R0)N1, requiring postoperative chemoradiotherapy after definitive resolution of the catecholaminergic hypersecretion.

## Treatment

Therefore, approximately eight months after the emergency department admission and one month after lumpectomy, she underwent simultaneous right adrenalectomy and lower parathyroidectomy with no intraoperative complications after adequate alpha- and beta-adrenergic blockade plus a hypersaline diet and oral fluid administration (Fig. 2D). The postoperative period was uneventful. The blood pressure remained controlled, in association with the normalization of the plasmatic metanephrines and PTH values, consistent with the surgical resolution of the pheochromocytoma and PHPT (Table 3). After that, the patient performed 25 sessions of radiotherapy and 5 months of chemotherapy with anthracycline, trastuzumab, and paclitaxel.

**Table 3:** Analytical parameters revealing resolution of catecholamine hypersecretion and primary hyperparathyroidism after right adrenalectomy and parathyroidectomy.

Parameter	Result	Reference value
Calcium and phosphate metabolism		
PTH, pg/mL	37.83	18.5–88
Total calcium, mg/dL	9.3	8.3–10.6
Corrected calcium, mg/dL	8.9	8.3–10.6
Albumin, g/dL	4.5	3.4–5.4
Plasma		
Epinephrine, pg/mL	11	<60
Norepinephrine, pg/mL	39	<650
Dopamine, pg/mL	8	<30
Metanephrine, pg/mL	31	<65
Normetanephrine, pg/mL	89	<196
Urine (volume = 2,300 mL/24 h)		
3-Methoxytyramine, μg/mL	264	<434

PTH, parathyroid hormone.

## Outcome and follow-up

The patient was evaluated in the specialized cardiomyopathy cardiology clinic and was discharged after showing clinical improvement. Follow-up dual-energy X-ray absorptiometry, performed 3 years after parathyroidectomy, demonstrated an improvement in bone mineral density, with T-scores of −2.9 at the lumbar spine, −1.6 at the femoral neck, and −2.6 at the distal radius, remaining within the osteoporotic range but showing a significant improvement compared with previous measurements.

Currently, she is under hormone therapy with the breast carcinoma in remission and is being followed up by the endocrinology and medical oncology departments.

## Discussion

TS is a reversible LV dysfunction that shares common features with acute coronary syndrome and is thought to be caused by catecholamine-mediated injury (5). Current evidence suggests that TS is typically sporadic; however, genetic susceptibility with a likely polygenic background is increasingly recognized. Variants affecting adrenergic signaling and cardiomyopathy-related genes may contribute to disease susceptibility, although no definitive genetic markers or Mendelian inheritance pattern have been identified. A direct molecular association between TS and NF1 has not been demonstrated; nevertheless, NF1-related vasculopathy and endothelial dysfunction could theoretically increase susceptibility to catecholamine-induced myocardial dysfunction (8). TS is triggered by a precipitating factor (emotional, physical, or combined) identified in 70% of cases, and among the physical trigger factors that may induce TS are PPGLs (5). A hallmark of PPGL-induced TS is the increased prevalence of apical sparing pattern, which occurs in 35% (inverted 30% and midventricular 5%) of patients, when compared to 2.2% in all TS cases. There is a lower incidence of the typical apical ballooning pattern, and in the majority of cases, left ventricular ejection fraction (LVEF) is markedly decreased. PPGL-induced TS has a high complication rate (68%) – although the reported in-hospital mortality rate is relatively low (2.5%) – and a higher recurrence rate (18%), being the early diagnosis of both conditions crucial to a good prognosis (4, 8).

Regarding PPGL-induced TS management, the presence of LV outflow tract obstruction or progression to cardiogenic shock guides the approach. However, it should be emphasized that the use of beta-blockers is contraindicated in PPGLs in the absence of alpha-adrenergic blockage, as occurred with our patient, due to unopposed stimulation of alpha-receptors and the imminent risk of hypertensive crisis. The definitive treatment of PPGLs is the tumor resection, being the uptitration of alpha-adrenergic antagonists crucial in the perioperative period (4, 8).

The prevalence of PPGLs in patients with NF1 is highly variable, depending on the type of study carried out, but may reach up to 50% in hypertensive patients. Its classic manifestation in the form of paroxysms of palpitations, headache, hyperhidrosis, and arterial hypertension, similar to what was seen in our patient, occurs in 33–58% of the patients; however, the occurrence of TS is very rare in this context (3, 4).

The co-occurrence of PPGLs and PHPT in NF1 is infrequent, with only a dozen case reports in the literature to date, mimicking MEN 2A syndrome (7). The presence of PHPT in patients with NF1, usually in association with a solitary parathyroid adenoma, acquires special relevance in these patients, who, by themselves, may already present some degree of bone dysplasia, and, due to PTH-induced bone resorption, makes this bone even more vulnerable (9). For this reason, although PHPT is uncommon in NF1, it would perhaps be pertinent to exclude this diagnosis by screening for hypercalcemia in all patients with NF1 (especially considering the low cost and ease of this measurement), thus reducing the morbidity that the diagnosis and late treatment may entail.

This is, as far as we know, the first case described in the literature of a patient with NF1 and a synchronous diagnosis of pheochromocytoma, PHPT, and breast carcinoma. Indeed, NF1 patients are at an increased risk of malignancy and have a life expectancy of about 10–15 years shorter when compared to the general population, being the mortality primarily due to cardiovascular diseases and malignant neoplasms (10).

In conclusion, this rare case highlights the relevance of a high level of suspicion of all the nosological entities that can be associated with NF1 in order to allow an early diagnosis. Even the less frequent ones, such as PHPT, which, when in association with PPGLs, should lead us to the differential diagnosis with MEN2. Furthermore, it also emphasizes the importance of a multidisciplinary approach, allowing a good prognosis for the patients and their families, as it allows a prompt screening of NF1 patients’ family.

## Declaration of interest

The authors declare that there is no conflict of interest that could be perceived as prejudicing the impartiality of the research reported.

## Funding

This research did not receive any specific grant from any funding agency in the public, commercial, or not-for-profit sector.

## Patient consent

Written informed consent for the publication of their clinical details and clinical images was obtained from the patient.

## Author contribution statement

NF, VL, and TP wrote the case. OA, AL, ADSL, and CMM supervised the writing process and validated the final version of the manuscript. NF, VL, TP, OA, AL, and ADSL helped in the case management, with CMM being the main physician of the patient.
